# The role of NURR1 in metabolic abnormalities of Parkinson’s disease

**DOI:** 10.1186/s13024-022-00544-w

**Published:** 2022-06-27

**Authors:** Murad Al-Nusaif, Yuting Yang, Song Li, Cheng Cheng, Weidong Le

**Affiliations:** 1 Center for Clinical Research on Neurological Diseases, the First Affiliated Hospital, Dalian Medical University, Dalian, 116021 China; 2 Liaoning Provincial Key Laboratory for Research on the Pathogenic Mechanisms of Neurological Diseases, the First Affiliated Hospital, Dalian Medical University, Dalian, 116021 China; 3 Institute of Neurology, Sichuan Academy of Medical Science, Sichuan Provincial Hospital, Chengdu, 610072 China

**Keywords:** NURR1, Dopaminergic neuron, α-Synuclein, Parkinson’s disease, Metabolism, Mitochondria

## Abstract

A constant metabolism and energy supply are crucial to all organs, particularly the brain. Age-dependent neurodegenerative diseases, such as Parkinson’s disease (PD), are associated with alterations in cellular metabolism. These changes have been recognized as a novel hot topic that may provide new insights to help identify risk in the pre-symptomatic phase of the disease, understand disease pathogenesis, track disease progression, and determine critical endpoints. Nuclear receptor-related factor 1 (NURR1), an orphan member of the nuclear receptor superfamily of transcription factors, is a major risk factor in the pathogenesis of PD, and changes in NURR1 expression can have a detrimental effect on cellular metabolism. In this review, we discuss recent evidence that suggests a vital role of NURR1 in dopaminergic (DAergic) neuron development and the pathogenesis of PD. The association between NURR1 and cellular metabolic abnormalities and its implications for PD therapy have been further highlighted.

## Background

Parkinson’s disease (PD) is the second most common neurodegenerative disease, which has increased from 2.5 million cases in 1990 to over 6 million cases in 2016 [1, 2]. The number of people at risk for developing PD is predicted to rise to 14.2 million by 2040 [3]. Environmental factors in genetically predisposed individuals are thought to contribute to the pathogenesis of this multifaceted disease [4]. However, multiple mechanisms and pathway dysfunctions accelerate the pathogenesis of PD, including oxidative stress, mitochondrial dysfunction, cellular calcium (Ca^2+^) imbalance, neuroinflammation, and other neurotransmitter system deficits [5, 6]. The main neuropathological hallmarks of PD include the substantial loss of dopaminergic (DAergic) neurons within the pars compacta of the substantia nigra (SNpc), and the development of intracytoplasmic α-synuclein-containing Lewy bodies, resulting in diminished facilitation of voluntary movement [7–9].

Cellular metabolic status and mammalian gene expression interact under the critical regulation of various signaling pathways, transcription factors (TFs), and epigenetic remodelers that affect cell fate during development [10–13]. Each cell type in the brain has a distinct metabolic profile and sustained cellular function over time; however, only a limited degree of metabolic flexibility can react to external stimuli [14, 15]. Cellular metabolic changes that exceed an adaptability threshold will endanger brain cellular resistance and function. Metabolic alterations in protein, glucose, lipid, and dopamine (DA) have been noticed in PD [16–18]. Growing evidence demonstrates that PD patients have altered unsaturated fatty acids (FAs), bile acid, steroid hormones, glucose, and amino acid metabolisms [18–21]. Changes in DA-related metabolites in PD-derived midbrain DAergic neurons have recently been reported, along with a significant increase in the expression of the DA-related genes, such as phenylalanine hydroxylase, tyrosine hydroxylase (TH), catechol-O-methyltransferase, and monoamine oxidase A and B [17]. These findings suggest that exploring the metabolic abnormalities is imperative in understanding PD’s pathogenic mechanism.

TFs bind at a particular location and time to a specific gene sequence and regulate the expression of the target gene, allowing them to control the development processes of the cells. Nuclear receptor-related factor 1 (NURR1) is one of these TFs required for the differentiation, maturation, and maintenance of DAergic neurons during their development [22, 23]. In the present review, we first build a framework for NURR1 involvement in PD and then detail the participation of NURR1 in modulating metabolic states and individual metabolites to control the epigenetic landscape and cellular identity. Overall, understanding the roles of NURR1 in cellular metabolic abnormalities in PD could be crucial for developing timely and tolerable NURR1-targeting modalities for PD therapy.

## Role of nuclear receptor-related factor 1 in dopaminergic neuron development and Parkinson’s disease

The nervous system is developed through the tremendous proliferation and differentiation of neural stem cells. Their fate is determined by precise signal patterning and TFs that allow for the correct regionalization, differentiation, and functional integration of new cells [24, 25]. NURR1, as a member of the “zinc finger” TF superfamily, is expressed early in embryogenesis at E10.5 and is detectable throughout the life [26, 27]. The nuclear receptor 4A (NR4A) subfamily is comprised of three nuclear receptors: NUR77 (NR4A1), NURR1 (NR4A2), and NOR1 (NR4A3). Although the deoxyribonucleic acid (DNA) binding domains in the three family members of NR4A have high similarity, their biological functions are quite different [28–31]. Many organs and tissues, including the brain, pancreas, liver, muscles, and fat, express the NR4A family members [31–35]. NURR1 is found in various central nervous system regions, including the cortex, hippocampus, brain stem, spinal cord, and olfactory bulb [36, 37]. The *Nurr1*-deficient (*Nurr1*^−/−^) mice are unable to develop midbrain DAergic neurons and die shortly after birth [38]. Those mice show impaired motor function and significant DAergic neuron loss in the SNpc and ventral tegmental area (VTA) [39]. The expression of NURR1 in the midbrain DAergic neurons decreases with age, which coincides with the increased morbidity of PD [40, 41].

NURR1 and its transcriptional targets were downregulated in DAergic neurons with a high level of the disease-causing protein α-synuclein in the midbrain [42]. In vitro, α-synuclein-induced activation of protein phosphatase 2A leads to inhibition of the phosphorylation activity of TH and aromatic amino acid decarboxylase (AADC) [43]. Furthermore, DA content is reduced in cells transfected with the A53T mutant α-synuclein [43, 44]. Previous research in the PD mouse model found that *Nurr1* is co-localized with TH^+^ neurons in the SNpc, VTA, retrorubral field, and olfactory bulb [45]. NURR1 interacts with other factors to regulate the expression of TH, AADC, and vesicular monoamine transporter 2 (VMAT2), which are essential in the synthesis, storage, and release of DA [46–49]. There is a significant correlation between NURR1 activity and the stabilization of the NURR1 ligand-binding domain (LBD) [50]. Additionally, NURR1 is a critical factor for the specification of DA neurotransmitter identity by activating TH gene transcription [51, 52]. *NURR1* mutations and polymorphisms that cause either reduced expression or dysfunction have been linked to familial and sporadic PD [53]. The determinant roles of NURR1 in the DAergic neuron genesis and PD development lead to an opportunity to develop novel therapeutics.

## Cellular metabolism changes in Parkinson’s disease and potential roles of nuclear receptor-related factor 1

Neural cells are prone to cellular metabolic abnormalities due to their high specialization and reliance on metabolic balance. Here, we highlight NURR1’s role in the metabolic abnormalities associated with PD.

### The impact of nuclear receptor-related factor 1 in α-synuclein-mediated dopamine cellular metabolism impairment

α-Synuclein is a 140 amino acid protein that is ubiquitously expressed in the brain, particularly throughout the neocortex, hippocampus, SNpc, thalamus, olfactory bulb, and cerebellum, where it plays a crucial role in synaptic function and plasticity [54–57]. α-Synuclein in presynaptic terminals is unfolded but misfolds under certain conditions [55, 58]. α-Synuclein is a well-established presynaptic protein [57, 59], and it is initially described as a nuclear protein [60, 61]. Various α-synuclein species have been identified in the nucleus of neuronal cells from the brains of PD patients [62–64] and in the animal and cellular models of PD [62, 65, 66]. Furthermore, α-synuclein significantly affects transcription and other cellular processes such as DNA integrity and the ER-Golgi system [67, 68]. In vitro, Outeiro et al. observed a reduction in toxicity of accumulated high molecular weight α-synuclein species after relocating them in the nucleus [69]. Thus, the α-synuclein nuclear localization, phosphorylation, and transcriptional regulation via DNA binding may be critical for cell homeostasis, division, and differentiation. Unni et al. also demonstrated that α-synuclein modulates DNA repair, suggesting that cytoplasmic α-synuclein aggregation may cause a loss of function, leading to increased DNA double-strand breaks and neuronal programmed cell death [70].

α-Synuclein is involved in several steps required to trigger exocytosis in the presynaptic terminal [57]. Under pathological conditions, toxic α-synuclein species trigger the dysregulation of several synaptic proteins, leading to functional impairment of the presynaptic terminal in the brains of animal models of PD and PD patients [71–73]. As previously stated, α-synuclein not only negatively modulates the activity of enzymes responsible for DA synthesis, but also impairs the transport and uptake of DA by altering the activity of VMAT2 and the DA transporter (DAT) [74–78]. The interaction of α-synuclein with surface DAT affects transporter function, which can change the synaptic availability of DA and substantially affect the membrane microenvironment near the transporter, which may impact DA neuron homeostasis [79]. These events may lead to a cycle of α-synuclein accumulation and dysregulated DA that leads to synaptopathy and neurodegeneration.

We have demonstrated that mutations in the first exon of *NURR1* (−291Tdel and − 245 T → G) are linked to familial PD [53]. Furthermore, we found that NURR1 expression was significantly reduced in the PD postmortem SNpc tissues with α-synuclein inclusions compared to age-matched healthy controls (HC) [42]. NURR1 expression was normal in the SNpc neurons without inclusions and in the hippocampal neurons of PD patients, demonstrating that this change is region-specific [42]. A decrease in SNpc NURR1 expression was also observed in some progressive supranuclear palsy and Alzheimer’s disease patients [42], indicating that reducing NURR1 in DAergic neurons is linked to intracellular pathology in synucleinopathies and tauopathies. NURR1 expression in the SNpc of α-synuclein homozygous mice significantly decreased with age [80]. Similar findings were achieved when the α-synuclein preformed fibril was injected into the putamen of non-human primates [81]. Furthermore, *NURR1* expression was decreased in PD patients’ peripheral blood lymphocytes (PBLs) compared to HC and neurological disease controls [82]. The reduction in *NURR1* expression in PBLs suggests systemic involvement of NURR1 in PD, which might potentially be used to identify patients with PD associated with central DAergic system impairments [82].

In addition to the critical role in the developing and reprogramming DAergic neurons, NURR1 has been shown to preserve and protect DAergic neurons in several animal and cellular models of PD [81, 83–85]. Furthermore, bexarotene, a retinoid X receptor (RXR) ligand that forms heterodimers with NURR1, has been demonstrated to co-regulate NURR1 target genes, including the TH receptor component [86]. This is supported by the finding that the induction of *Nurr1* expression in primary DAergic neurons expressing α-synuclein restores the dysregulated gene functions [86].

**Fig. 1:**
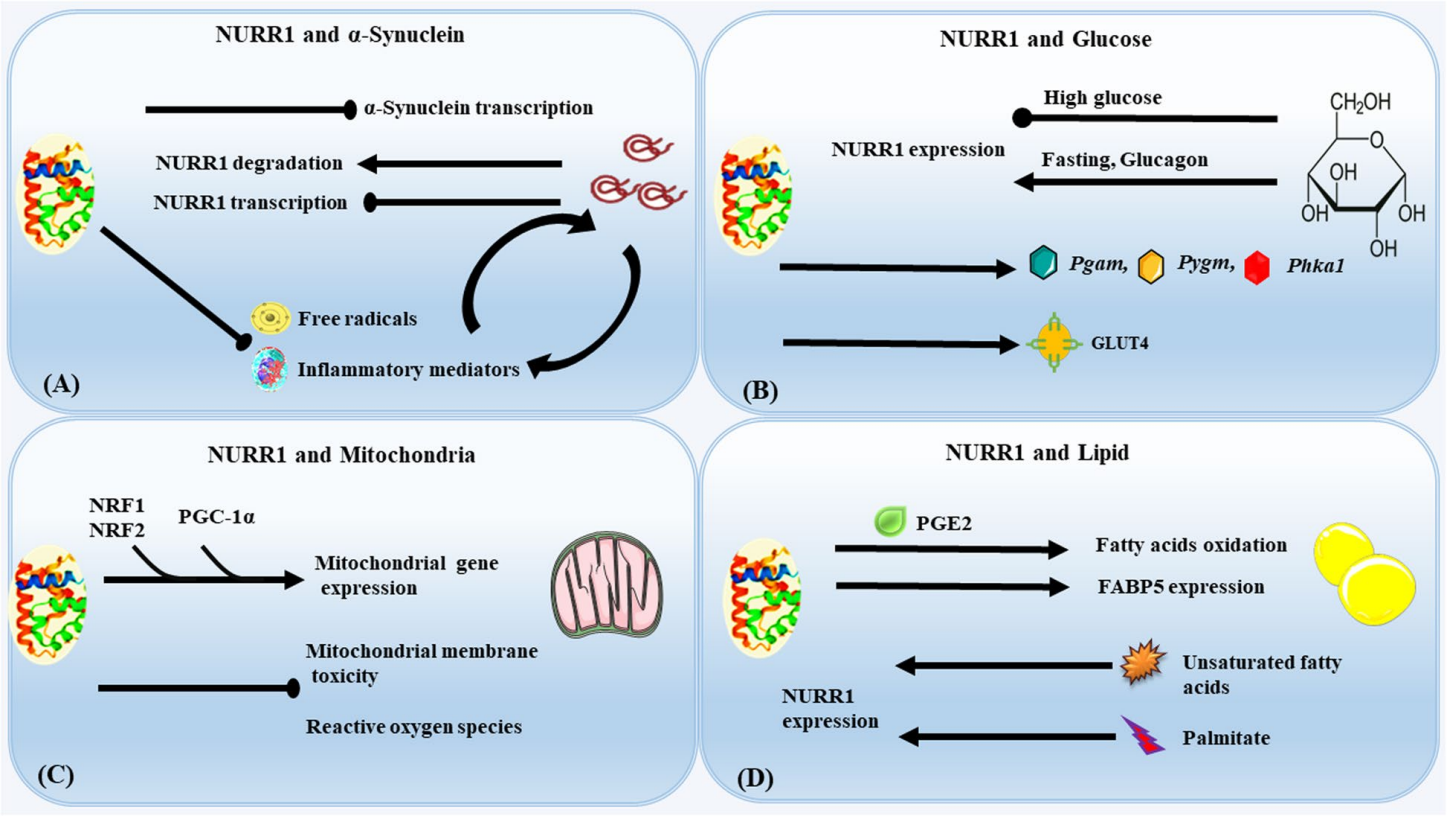
NURR1 roles in the metabolism of α-synuclein, lipids, glucose, and mitochondria. Sharp arrows (positive regulation), rounded arrows (negative regulation) (**A**) α-Synuclein, and NURR1 have a detrimental impact on each other. α-Synuclein promotes inflammatory mediators and free radicals, and they, in turn, exacerbate α-synuclein accumulation, creating a vicious cycle, and NURR1 could interrupt this vicious cycle. **B** NURR1 activates GLUT4 transcription and induces genes involved in glucose and glycogen metabolism; Simultaneously, NURR1 expression could be inhibited by high glucose. Fasting and glucagon treatment induce *Nurr1* expression (**C**) NURR1 in DAergic neurons positively regulates many nuclear-encoded mitochondrial genes and protects cells against the mitochondrial membrane and reactive oxygen species (**D**) Activating NURR1 promotes the oxidation of FAs, also up-regulates FABP5 expression. Furthermore, unsaturated FAs activate transcriptional function of NURR1. NURR1 expression and nuclear translocation are increased in response to a lipotoxic insult of palmitate. Abbreviations: NURR1: Nuclear receptor-related factor 1; FABP5: Fatty acid-binding protein 5; GLUT: Glucose transporter; *Pygm*: Phosphorylase glycogen muscle; *Phka1*: Phosphorylase kinase α 1; *Pgam*: Phosphoglycerate mutase 2; PGE2: Prostaglandin E2; NFR1,2: Nuclear respiratory factors 1 and 2; PGC-1α: Peroxisome proliferator-activated receptor-gamma coactivator1-alpha

Our recent work has demonstrated that α-synuclein can modulate the transcription activity of the *Nurr1* promoter region, between − 605 and − 418 bp, which contains the nuclear factor kappa B (NF-κB) binding site [87]. Furthermore, overexpression of α-synuclein (wild type or A53T mutant) reduces the NF-κB binding quantity to the *Nurr1* promoter, resulting in decreased transcription of *Nurr1* [87] and potentially inducing proteasome-dependent NURR1 degradation in the midbrain DAergic neurons [88]. Overexpression of α-synuclein inhibits NF-κB expression and increases glycogen synthase kinase 3β (GSK-3β) protein levels in the DAergic neurons, implying that the pathological effects may be mediated by the NF-κB signaling pathway [89]. Ji et al. found that prostaglandin E2 (PGE2) stimulation of E-type prostaglandin receptor 1 upregulates the expression of *NURR1* via the activation of NF-κB signaling pathways [90], indicating that α-synuclein can suppress the expression of endogenous NURR1. Interestingly, NURR1 has a considerable inhibitory effect on α-synuclein transcription [91]. Therefore, NURR1 and α-synuclein may regulate each other (Fig. 1A).

The interplay of Ca^2+^, cytosolic DA (DAcyt), and α-synuclein contribute to the selective vulnerability of SNpc neurons in PD [92], and DAergic neurons in SNpc also depend on Ca^2+^ channel pacemaking [93, 94]. Therefore, various strategies for preventing neuronal death in PD can potentially be employed, including inhibiting Cav1.3 channel activity [95] and blocking the toxicity of DA-α-synuclein interactions [96]. Sulzer et al. found that levodopa (L-DOPA) increases DAcyt in the SNpc neurons 2 to 3-folds higher than VTA neurons. This response is dependent on dihydropyridine-sensitive L-type Ca^2+^ channels, resulting in greater susceptibility of SNpc neurons to L-DOPA-induced neurotoxicity [92]. Additionally, in hemiparkinsonian rats, Steece-Collier et al. demonstrated that genetic silencing of the striatal L-type Ca^2+^ channel prevented and reversed L-DOPA-induced dyskinesia (LID) [97]. Furthermore, Sellnow et al. found that ectopic induction of striatal NURR1 might induce LID behavior and associated neuropathology [98]. Therefore, NURR1 may play a crucial role in regulating the transcriptional and plasticity activities of Cav1.3 [98]. Cav1.3 activity, mediated by calcineurin, regulates NURR1 expression [99]. Moreover, NURR1 might be vital to modulate the interaction of DAcyt, Ca^2+^, and α-synuclein, thereby avoiding the selective death of SNpc neurons [92, 97–100]. Understanding this pathway may help identify drug targets and their future development to avoid neurotoxic and synaptic plasticity changes.

Neuroinflammation processes significantly contribute to PD pathogenesis. A meta-analysis [101] reported that a single-nucleotide variation within the human leukocyte antigen locus increases the risk of developing PD, implying an immune-related susceptibility. Furthermore, epidemiological studies [102] revealed a negative correlation between the incidence of PD and the use of anti-inflammatory medications, particularly nonsteroidal anti-inflammatory drugs, supporting the hypothesis that inflammation may promote underlying PD processes. α-Synuclein can activate microglia, produce inflammatory mediators, and trigger oxidative stress [103–105]. The generation of inflammatory mediators and increased levels of free radicals can exacerbate α-synuclein accumulation, producing a vicious cycle of continuous progression in PD pathogenesis (Fig. 1A) [105–107]. Microglia, astrocytes, and macrophages express *Nurr1* mRNA and NURR1 protein under basal conditions and can elevate *Nurr1* levels when activated [103, 108–110].

According to the report by Sajiao et al., NURR1 protects DAergic neurons from neurotoxicity and inflammation via inhibiting the expression of proinflammatory mediators in microglia and astrocytes (Fig. 1A) [111]. NURR1 is also a vital component of a negative feedback loop in microglia and astrocytes by recruiting a corepressor for element-1–silencing transcription factor to NF-κB target genes [111]. They reported that TH^+^ DAergic neurons’ survival rate decreased in response to inflammatory stimuli during *Nurr1*-deficiency [111]. Oh et al. found that NURR1 may exert its anti-inflammatory effects through modulating the expression of RAS guanyl-releasing protein 1 in lipopolysaccharide (LPS)-induced inflammatory cell model [112]. Enhancing NURR1 expression has been reported to reduce oxidative stress and protect DAergic neurons by decreasing apoptosis-related proteins and increasing antioxidant proteins [113, 114]. As a result, scavenging free radicals and regulating the generation of inflammatory mediators is one of the keys to preventing and delaying the progression of PD.

These findings imply that synuclein pathological changes may be driven by at least part of the cellular metabolic pathogenesis of PD. NURR1 is one of the targets directly or indirectly affected by the inclusion bodies’ pathological changes in PD. NURR1 may potentially protect DAergic neurons from α-synuclein in various ways, thereby delaying or blocking the progression of PD neuropathology.

### Regulating effects of nuclear receptor-related factor 1 in the altered energy metabolism of Parkinson’s disease

The brain represents ~ 2% of the bodyweight of the average adult human, but it consumes 20% of the daily energy source [115, 116]. Given that one of the common features of PD pathogenesis is an energy deficit and decreased adenosine triphosphate (ATP) levels, oxidative phosphorylation and glycolysis could be critical pathological and therapeutic avenues for this neurodegenerative disease [117–121]. Neurons primarily rely on oxidative phosphorylation in mitochondria to meet such energy needs, with glycolysis accounting for a tiny portion of their energy supply [122]. Even though PD’s main metabolic pathological drive is from SNpc oxidative phosphorylation, there is increasing information on PD’s cortical brain metabolic pathology [123–125]. DA-deficiency in the SNpc causes an imbalance in both the indirect (inhibitory) and direct (activating) pathways of the cortico–basal ganglia–cortical circuit, which may lead to hypokinesia in PD [126–129]. Consequently, several imaging studies have demonstrated changes in brain metabolism in PD, and in the early stages of the disease, the cortex may exhibit a widespread low-metabolic state [130, 131]. Furthermore, lipoxidation, glycoxidation, and lipid peroxidation markers are elevated in the cerebral cortex of PD patients [132].

Human SNpc DAergic neurons have an exuberant and highly arborized axonal arborization, with upwards of a million neurotransmitter release sites per SNpc DAergic neuron [133, 134]. This characteristic can potentially inflict a significant bioenergetic load on these cells and subject them to a progressive elevation of oxidative stress [135, 136]. Additionally, the study by Giguère et al. found that SNpc DAergic neurons have 2-fold greater axonal arborization and are more susceptible to a 6-hydroxydopamine (6-OHDA) lesion in mice with the selective deletion of DA D2 receptor [137]. Several studies have implicated mitochondrial dysfunction, and cellular bioenergetic alterations as an underlying cause of PD [138, 139]. DAergic neurons demand more energy than other neuronal cell types [140, 141], rendering them more susceptible to mitochondrial dysfunction and, ultimately, cell death [142, 143]. Defects in mitochondrial respiration are corroborated by lower glucose consumption in PD patients [144] and lower pyruvate oxidation in PD patients’ fibroblasts [145], indicating lower acetyl-CoA entry into the tricarboxylic acid (TCA) cycle [144]. Mitochondrial respiration abnormalities may inhibit complex I nicotinamide adenine dinucleotide (NADH)-ubiquinone reductase of the electron transport chain (ETC), which might play a role in the pathogenesis of PD [146].

#### Role of nuclear receptor-related factor 1 in glucose metabolism of Parkinson’s disease

Glycolysis is one of the primary processes that glucose provides energy, and abnormal glucose metabolism is critical in PD’s molecular mechanism [147, 148]. Overexpression of α-synuclein along with paraquat exposure leads to increased glucose accumulation, impaired glycolysis activity, and mitochondrial respiration [149]. Glucose transporter (GLUT) inhibition prevents α-synuclein from potentiating paraquat toxicity. Furthermore, inhibition of the pentose phosphate pathway (PPP) protects against this synergistic toxicity [149]. Apart from its essential role in antioxidant defense and nucleic acid synthesis, PPP provides nicotinamide adenine dinucleotide phosphate for fatty acid and cholesterol biosynthesis [149–151].

Low expression of PPP enzymes and an inability to build antioxidant reserves are the early events in the development of sporadic PD, and mitochondrial damage in PD may be a direct result of PPP dysregulation [151]. α-Synuclein plays a vital part in altered glucose metabolism by PPP [151]. Glucose-6-phosphate dehydrogenase (G6PD), the rate-limiting enzyme of PPP, is found throughout the body, and its expression and activity vary over a 10-fold range, with the highest level seen in the brain [152, 153]. The expression and activity of G6PD were increased when LPS was used in vitro and in vivo PD models, and this increase is linked to microglial activation and DAergic neurodegeneration [150]. G6PD knockdown or inhibition reduced the LPS-induced reactive oxygen species (ROS) over-production and NF-кB activation, thereby reducing microglial activation [150, 154]. These findings demonstrate that G6PD has a role in PPP dysfunction and neuroinflammation, leading to DAergic neurodegeneration.

Epidemiological evidence suggests a link between diabetes and PD, with hyperglycemia as one of the factors in neurodegeneration of the nigrostriatal pathway in PD [155, 156]. In addition to hyperglycemia, emerging evidence implies that insulin resistance in the brains of PD patients and impaired brain insulin signaling are potential contributors to PD pathogenesis [157]. In support of this, there is a downregulation of the insulin receptor in the SNpc and an increase in insulin resistance in patients with PD [158–160]. The activation of insulin signaling can modulate the degradation of α-synuclein and inhibit α-synuclein fibril formation by activating the insulin-degrading enzyme [161], which is supported by the fact that reversing insulin resistance can prevent the α-synuclein-induced toxicity [162]. In agreement with the effect of insulin signaling, postmortem analysis found that protein kinase B or Akt (PKB/AKT), a serine/threonine kinase, decreased in PD patients’ brains [163]. The inhibition of AKT signaling exaggerates DAergic cell death [164, 165], providing a further mechanistic link between impaired insulin signaling and PD. AKT phosphorylates NURR1 at Ser^347^, increasing protein stability [166]. Thus, the defective insulin signaling appears to be at the crux of insulin resistance and PD pathogenesis.

High glucose exposure in a mouse model of diabetes reduces NURR1 expression and nuclear translocation in Müller cells [167]. On the other hand, NURR1 agonists inhibit Müller cell activation and retinal ganglion cell loss [167, 168]. Furthermore, downregulation of NURR1 promotes high glucose-induced Müller cell activation by upregulating the NF-κB/Nucleotide-binding oligomerization domain-like receptor protein 3 (NLRP3) inflammasome axis [167, 169]. NURR1 agonists may have significant anti-inflammatory and neuroprotective effects on Müller cells in diabetic retinopathy [167]. Expression of NURR1 and GSK-3β are downregulated in the peripheral blood mononuclear cells (PBMCs) of type-2 diabetes (T2D) patients [168]. Furthermore, high levels of proinflammatory cytokines and low NR4A expression cause insulin resistance by inhibiting the expression of GLUT and the phosphorylation of insulin receptors [168]. Long-term insulin resistance contributes to hyperglycemia and hyperlipidemia, further downregulating NURR1 expression and resulting in a vicious cycle during T2D pathogenesis [168]. Furthermore, NLRP3 inflammasome activation increases in patients with T2D [170]. Therefore, the NURR1/NF-κB/NLRP3 inflammasome might be a potential pathway by which NURR1 regulates glucose metabolism, which is still an open field for research.

NURR1 activates *GLUT4* transcription in skeletal muscle, and NURR1 overexpression strongly induces the expression of genes involved in glucose and glycogen metabolism, such as phosphorylase glycogen muscle, phosphorylase kinase α 1, and phosphoglycerate mutase (Fig. 1B) [171]. *Nurr1* overexpression in skeletal muscle enhances glucose uptake, utilization, and storage. In contrast, fasting and glucagon treatment induces *Nurr1* expression in hepatic cells [32, 172]. Treatment of the PBMCs with high glucose and palmitic acid inhibits *NURR1* expression in a dose- and time-dependent manner [168]. Similarly, the NURR1 agonist, amodiaquine, enhanced glucose tolerance and restored insulin levels to normal in obese mice [32]. Although the underlying mechanisms are unknown, NURR1 plays a function in the physiological process of glucose metabolism that helps protect the DAergic neuron from the detrimental consequences of metabolic disturbances, thereby preventing cell death. In addition, it may promote searches to find novel therapeutic targets from a metabolic perspective.

#### Association of nuclear receptor-related factor 1 in mitochondrial dysfunction of Parkinson’s disease

It is well understood that mitochondria play a vital role in aerobic glycolysis. Mitochondria are the cells’ energy producers and are critical intercellular linkers with other organelles. Mitochondria control energy metabolism, biosynthesis, immunological response, and cell turnover by interacting with the endoplasmic reticulum, peroxisomes, and nucleus through signal transduction, vesicle transport, and membrane contact sites [173]. ETC is a critical component of mitochondrial energy production. During oxidative phosphorylation, NADH provided by the TCA cycle is oxidized and provides electrons to the ETC [174, 175]. Metabolic alterations and inactivation of the ETC complex are characteristics of PD; thus, poor energy metabolism is linked to PD [16, 149]. A large meta-analysis of genome-wide gene expression studies has reported that genes encoding oxidative phosphorylation proteins correspond to the functional category of most of the deregulated genes in the remaining DAergic neurons in PD [176]. NURR1 works with numerous genes associated with the DAergic neuron phenotype, including DA metabolism, neurotransmission, axonal development, mitochondrial function, and cell survival [177–179].

NURR1 regulates numerous nuclear-encoded mitochondrial genes positively, with over 90% of the genes creating down-regulated respiratory chains in *Nurr1*-ablated DAergic neurons [179]. It has been proposed that decreased NURR1 activity is linked to mitochondrial malfunction, which accelerates neurodegeneration in PD [180]. Furthermore, NURR1 regulates various proteins that play a role in mitochondrial functions, including pituitary homeobox 3 and Wnt/β-catenin, which regulate DAergic neurogenesis [22, 85, 181–183]. On the other hand, several studies have demonstrated that NURR1 protects cells by regulating mitochondrial genes such as sodium oxide dismutase 1 and mitochondrial translation elongation factor [177]. Recent research has also revealed that NURR1 could protect cells against the potential toxicity of mitochondrial membrane and intracellular ROS (Fig. 1C) [184].

Peroxisome proliferator-activated receptor-gamma coactivator1-alpha (PGC-1α) and nuclear respiratory factors 1 and 2 (NRF1 and NRF2) are fundamental transcriptional regulators of energy metabolism, acting as suppressors of ROS in neurons [185–187], as well as critical regulators of nuclear-encoded mitochondrial genes [187, 188]. Increased methylation of PGC-1α in the SNpc of α-synuclein mice can lead to decreased PGC-1α expression and mitochondrial content [189]. Previous research has shown that PGC-1α can be induced by parathyroid hormone, resulting in coactivation of the *Nurr1* promoter activity in the osteoblast, suggesting another potential functional connection between NURR1 and PGC-1α, which may protect cells against the potential toxicity of oxidative stress derived from mitochondrial dysfunction [190].

Apart from fundamental roles in generating energy and the metabolism of lipids and amino acids, mitochondria is also a key player in maintaining Ca^2+^ homeostasis [191]. Identification of the molecular components of the mitochondrial Ca^2+^ uniport complex has provided crucial insight into the function mitochondrial Ca^2+^ influx plays in energy production under an increased workload and, paradoxically, in disease development, such as neurodegeneration [192, 193]. L-type Ca^2+^ channel activation is critical for spontaneous DAergic neuron pacemaking, which is then accompanied by sustained Ca^2+^ entry through L-type channels [194, 195]. Ca^2+^ entry via L-type channels increases mitochondrial oxidative stress, which is amplified by deglycase-1 gene deletion [196, 197]. Recent studies in PD zebrafish and drosophila models have shown that lowering high mitochondrial Ca^2+^ levels could improve neurodegeneration [198, 199]. To summarize, NURR1 may interact with other TFs essential for expressing nuclear respiratory genes, such as NRF1 and NRF2, or with the transcriptional coactivator PGC-1α, which serves as a master regulator of mitochondrial biogenesis and cellular respiration. Mitochondria and NURR1 metabolic involvement remain poorly understood; future studies are needed to determine the NURR1 metabolic pathways and the role of oxidative phosphorylation in NURR1-ablation and overexpression models.

### Involvement of nuclear receptor-related factor 1 in the altered lipid metabolism of Parkinson’s disease

The human brain has the second-largest lipid content after adipose tissue. Lipids help maintain brain activities, such as synaptic function, making it highly vulnerable to lipid metabolic disorders [200, 201]. Lipids are involved in a multitude of aspects of PD pathology, including unique cytotoxic interactions with α-synuclein [202, 203], mutations in enzymes involved in lipid metabolism genes that increase the risk of PD development [204, 205], lipid pathway alterations [206, 207], and lipid involvement in oxidative stress and inflammation [208]. Disruption of the lipid membrane is one potential mechanism of cytotoxicity. Studies have shown that the toxicity of α-synuclein and docosahexaenoic acid (DHA) oligomers to cells is partially due to the disruption of the integrity of the lipid membrane [20, 202]. Mutations in glucocerebrosidase and sphingomyelinase 1 lead to a loss of glucocerebroside function and an increase in α-synuclein aggregation, subsequently augmenting the development of PD [209, 210]. Changes in membrane lipids have been observed in both affected and unaffected regions of PD patients’ brains and various experimental models of PD [211, 212], implying that changes in lipid metabolism or metabolic pathways may precede the development of PD. αSynuclein has some structural similarities with the class A2 lipoproteins and fatty acid-binding protein (FABP), which may play an important role in lipid metabolism [213–215].

Fatty tissue highly expresses NR4A members during the early stages of adipocyte differentiation [216]. *Nurr1* is upregulated during extreme obesity and normalized after fat loss [217]. Furthermore, activated NURR1 can promote the oxidation of FAs to supply ATP, which could be regulated by PGE2, a critical transcriptional integrator that allows crosstalk between the PGE2 and FAs oxidation pathways [218]. Interestingly, Briand et al. found that palmitate lipotoxic insult increased NURR1 expression and nuclear translocation in the insulinoma cell line, Min6 [219], implying the involvement of NURR1 in at least some FAs induced transcriptional responses. These results contradict the previously mentioned study in which palmitic acid could reduce the expression of NURR1 in PBMCs from patients with T2D [168]. It is worth noting that Nurr1 overexpression in purified human islets decreased the expression of a cluster of genes that are involved in inflammation control [219]. Decreased NURR1 and GSK-3β phosphorylation expression levels in PBMCs were negatively correlated with interleukin-6 and tumor necrosis factor-α levels [168]; whether this downregulation results from a long-term adaptive and protective response to glucose homeostasis remains to be determined by studying tissue-specific gene knockouts. Furthermore, the unsaturated FAs can directly bind to NURR1 and activate its transcriptional function (Fig. 1D) [220, 221].

FABP, also known as intracellular lipid chaperon, may dictate the destiny of lipids that coordinate lipid trafficking and signaling and are intimately linked to metabolic and inflammatory pathways [222]. FABP5 and NURR1 are expressed in the mouse brain; however, they are not co-localized in basal conditions and are both induced in response to stress stimuli such as brain injury, seizure, or inflammation [110, 223–225]. In HEK293 cells, NURR1 increases retinoic acid levels by upregulating FABP5-induced signaling of peroxisome proliferator-activated receptors and activating DHA-induced RXR [223]. All these findings suggest that NURR1 can influence the signaling of other nuclear receptors by regulating the expression levels of FABP5. Moreover, in vivo and in vitro, HX600, a synthetic agonist of NURR1/RXR, can reduce microglia-expressed proinflammatory mediators and prevent inflammation-induced cell death [226]. As a result, NURR1/RXR may play a dual role in PD, providing both neuroprotection from inflammation and symptomatic relief through upregulation of TH, AADC, and guanosine-5′-triphosphate cyclohydrolase I transcription and an increase in striatal DA level [227].

Clinical and experimental evidence suggests that steroid hormones, such as estrogen [228], progesterone [229], and thyroid pituitary axis hormones [230, 231], have a role in the pathogenesis of PD. Additionally, NURR1 regulates the synthesis of hormones, including aldosterone in the adrenal cortex [232, 233], osteocalcin in osteoblasts [234], and lactotropes in the female pituitary [235]. Although previous research has suggested that NURR1 may regulate some hormone metabolisms, it is unclear if NURR1’s role in hormone metabolism impacts PD. Understanding the molecular mechanisms of their involvements in PD would enable researchers to better explore the pathometabolic processes and signalings, which may elucidate tailored therapeutic targets for this devastating disease.

## Nuclear receptor-related factor 1-targeting therapy for Parkinson’s disease

Despite the extensive research breakthroughs, the current treatments for PD are primarily symptomatic relief, and there are no therapies available that can prevent or delay disease progression. It will be a big challenge to develop disease-modifying and mechanism-based approaches, although several preclinical investigations of targeted molecular therapeutics, for example, have been conducted with encouraging findings [236, 237].

A growing body of evidence from in vitro and in vivo studies has demonstrated that NURR1-activating compounds, NURR1 agonists, and *Nurr1* gene therapy can enhance DA neurotransmission and inhibit the microglial and astrocytic production of neurotoxic mediators [111, 184, 238], thereby protecting DAergic neurons from cell injury [238–240]. Using cell-based assays, Kim et al. found that three NURR1 agonist compounds among food and drug administration-approved drugs sharing an identical chemical scaffold targeting the NURR1 LBD can be exploited as a potential mechanism-based neuroprotective therapy for PD [84]. Importantly, these compounds significantly alleviate behavioral abnormalities in the lesioned 6-OHDA rat model of PD without any inducing symptoms of dyskinesia-like behavior [84].

Moreover, NURR1 modulators targeting RXR and the Wnt/β-catenin pathway may enhance the effects of NURR1-based therapies in PD [86, 241–244]. In a subacute mouse model of 1-Methyl-4-phenyl-1,2,3,6-tetrahydropyridine hydrochloride-induced PD, the herbal extract consisting of Bupleuri Radix, Moutan Cortex Radicis, and Angelica Dahuricae resulted in recovery from movement impairment [245]. This herbal extract is shown to upregulate NURR1 expression and consequently increase DA level, DAergic neurons, and fibers in the nigrostriatal projection [245].

Although the implications of NURR1 in PD treatment have not yet been thoroughly evaluated, identifying its molecular mechanisms in DAergic neuron development and cellular metabolic function may eventually help to develop individualized treatments aiming at the restoration of functional integrity of disease-specific brain pathology and reverse the decline of DAergic function in PD. Another promising strategy is identifying selective and safe NURR1 agonists to support DAergic neuron functions and reduce neuroinflammatory activity.

## Conclusion and perspective

Even though research on the involvement of NURR1 in DAergic neurons began more than 20 years ago, a recent surge of evidence indicates that it plays a critical role in embryonic development and cellular metabolism. The altered metabolic state of PD patients may result in the downregulation of NURR1 expression, which increases the deposition of α-synuclein, and promotes the formation of abnormal cellular metabolism, thereby culminating in a vicious circle. The restoration or enhancement of NURR1 expression and function may disrupt this cycle, prevent cellular metabolic disorders, and delay the progression of PD. NURR1-related developmental and cellular metabolism modulation may provide crucial new therapeutic insight for PD. At the current stage of PD research, the exact mechanism of NURR1 in neuronal development, cellular metabolic disorders, and PD pathogenesis is still not fully understood, and future studies to clarify this are required.

## Data Availability

Not applicable.

## Abbreviations

PD: Parkinson’s disease
DA: Dopamine
DAergic: Dopaminergic
SNpc: Compacta of the substantia nigra
Ca^2+^: Calcium
TFs: Transcription factors
NURR1: Nuclear receptor-related factor 1
TH: Tyrosine hydroxylase
AADC: Aromatic amino acid decarboxylase
6-OHDA: 6-hydroxydopamine
VMAT2: Vesicular monoamine transporter 2
DAT: DA transporter
NR4A: Nuclear Receptor 4A
DNA: Deoxyribonucleic acid
LBD: Ligand-binding domain
VTA: Ventral tegmental area
NF-κB: Nuclear factor-kappa B
GSK-3β: Glycogen synthase kinase 3β
DAcyt: Cytosolic DA
L-DOPA: Levodopa
LID: L-DOPA-induced dyskinesia
LPS: Lipopolysaccharide
ATP: Adenosine triphosphate
PBMCs: Peripheral blood mononuclear cells
HC: Healthy control
RXR: Retinoid X receptor
*PGE2*: Prostaglandin E2
GLUT: Glucose transporter
PPP: Pentose phosphate pathway
G6PD: Glucose-6-phosphate dehydrogenase
ROS: Reactive oxygen species
T2D: Type-2 diabetes
NLRP3: Nucleotide-binding oligomerization domain-like receptor protein 3
ATP: Adenosine-5′-triphosphate
ETC: Electron transport chain
NADH: Nicotinamide adenine dinucleotide
PGC-1α: Peroxisome proliferator-activated receptor-gamma coactivator1-alpha
NRF1 and NRF2: Nuclear respiratory factors 1 and 2
DHA: Docosahexaenoic acid
FAs: Fatty acids
TCA: Tricarboxylic acid
FABP5: Fatty acid binding protein 5
